# Growth performance and muscle histological characteristics of IPB-D3 chickens reared under intensive and free-range systems

**DOI:** 10.14202/vetworld.2026.282-294

**Published:** 2026-01-25

**Authors:** Andhika Yudha Prawira, Rizki Fitrawan Yuneldi, Isyana Khaerunnisa, Ahmad Furqon, Dwi Lestari, Ni Luh Putu Rischa Phadmacanty, Cahyo Budiman, Cece Sumantri

**Affiliations:** 1 Research Center for Applied Zoology, Research Organization for Life Sciences and Environment, National Research and Innovation Agency (BRIN), Bogor, Indonesia; 2 Research Center for Animal Husbandry, Research Organization for Agriculture and Food, National Research and Innovation Agency (BRIN), Bogor, Indonesia; 3 Department of Animal Production and Technology, Faculty of Animal Science, IPB University, Bogor, Indonesia; 4 Biotechnology Research Institute, Universiti Malaysia Sabah, Kota Kinabalu, Sabah, Malaysia

**Keywords:** animal welfare, food security, free-range rearing, IPB-D3 chicken, local genetic resources, muscle histology, SDG 12, SDG 2, sustainable poultry production

## Abstract

**Background and Aim:**

IPB-D3 chicken is a locally developed fast-growing composite line derived from Pelung, Sentul, Kampung, and Broiler strains. Despite its potential as a dual-purpose Indonesian breed, detailed information on its muscle histology, especially type IIX myofiber composition under different rearing systems, is lacking. This study aimed to evaluate the growth performance and muscle histological characteristics of IPB-D3 chickens reared under intensive and free-range systems.

**Materials and Methods:**

Ninety 12-week-old IPB-D3 chickens were reared for 12 weeks under two systems: intensive (n = 45) and free-range (n = 45). Samples of pectoralis major and quadratus femoris (Fem) muscles from 10 birds (five per group) were examined using hematoxylin-eosin, picrosirius red, and immunohistochemical staining for type IIX myofibers. Parameters such as fasciculus area, myofiber cross-sectional area, myofiber number per mm^2^, collagen percentage, and type IIX fiber intensity were analyzed using an independent t-test at a 95% confidence level (Statistical Package for the Social Sciences v.29.0).

**Results:**

No significant difference (p > 0.05) was observed in body weight or carcass yield between rearing systems. However, free-range chickens exhibited a significantly larger myofiber cross-sectional area and a higher proportion of high-intensity type IIX myofibers in the Fem muscle (p < 0.05), while the intensive system showed a higher percentage of intramuscular collagen (p < 0.05). The overall muscle morphology was similar between systems, with polygonal myofibers organized within collagen-bound fasciculi.

**Conclusion:**

This study provides the first histological characterization of IPB-D3 chickens, demonstrating that both rearing systems support comparable growth performance. Free-range rearing enhances thigh muscle hypertrophy and type IIX fiber development, whereas intensive rearing increases collagen deposition. These findings suggest that IPB-D3 chickens are adaptable to diverse production environments. Further studies should explore Myosin heavy chain gene expression, longitudinal muscle growth, and meat texture properties to improve sustainable rearing strategies and meat quality optimization for Indonesian local chicken development.

## INTRODUCTION

The growing consumer and market demand for chicken meat has accelerated innovations in developing local poultry breeds and strains with high productivity and rapid growth potential [1]. The superior genetic traits of indigenous Indonesian chickens provide a strong foundation for creating new composite breeds that meet community and market needs. Globally, poultry meat demand continues to rise, with production projected to increase by approximately 16% by 2025 [2]. According to Bakrie *et al*. [3], local chicken remains one of Indonesia’s most preferred livestock commodities due to its unique flavor and cultural acceptance. However, the productivity of local breeds is generally lower than that of commercial broilers, motivating the development of composite chickens to improve growth and carcass performance [1].

Composite chickens are the result of crossbreeding multiple breeds to combine desirable parental traits [4, 5]. The IPB-D3 line was developed through a cross between male Pelung-Sentul and female Kampung-Broiler (Cobb parent stock) chickens, with each parental strain contributing 25% of the genetic composition. Through successive selection and breeding, this composite line evolved into a fast-growing local chicken suitable for use as a parent stock [6]. At 12 weeks of age, IPB-D3 males can reach an average body weight of 1,880.5 g [7]. Despite this promising performance, further characterization is required to identify its superior attributes, particularly in muscle structure and growth-related genes.

The rearing system plays a crucial role in determining poultry product quality. As reported by Dal Bosco *et al*. [2], rearing conditions affect both physiological performance and meat characteristics. Increasing consumer awareness of animal welfare has driven interest in free-range systems that allow chickens to express natural behaviors, influencing not only welfare but also meat texture, structure, and safety perceptions [8]. Under-standing how rearing systems affect the muscle histology of IPB-D3 chickens is therefore vital for improving product quality and enhancing sustainability in local poultry production.

Growth and muscle performance can be evaluated using various parameters, including body and carcass weight, muscle strength, fascicle area, myofiber cross-sectional area, myofiber number per fascicle, and myofiber diameter in breast and thigh muscles. Additional texture-related indices, such as hardness, tenderness, springiness, chewiness, and fracture resistance, in pectoralis muscles provide complementary insights into meat quality [9–16].

The total number of myofibers formed prenatally, and their postnatal enlargement, determine final muscle mass, while myofiber characteristics influence the visual and textural attributes of meat [17]. Fast-growing birds typically have more myofibers than slow-growing birds [18]. The genetic composition of Indonesian composite chickens, being a mixture of parental traits, is expected to affect myofiber number, muscle size, fiber-type, and connective tissue distribution, all of which influence meat quality. Myofibers constitute the main structural component of chicken meat [19], with skeletal muscle primarily consisting of red (slow-twitch/type I) and white (fast-twitch/type II) fibers. The relative proportion of these fibers strongly affects key meat quality parameters, such as color, tenderness, and postmortem pH [20]. A higher proportion of type I and IIA fibers is generally associated with improved meat quality, whereas type IIX and IIB fibers are negatively correlated with desirable texture and sensory properties [21].

Previous histological studies [10, 11, 13–15, 22, 23] on the pectoralis muscle have been conducted in Pelung, Broiler, and Kampung chickens, and muscle performance has also been characterized in Sentul chickens [12]. However, similar evaluations have not yet been performed in IPB-D3 chickens. Given that IPB-D3 is a relatively new composite line, earlier studies have primarily addressed its growth performance, morphometric traits, reproductive capacity, nutritional responses, genetic characteristics, and immune functions [7, 24–31]. Therefore, detailed histological characterization remains essential to elucidate the muscle properties and adaptive potential of this promising Indonesian chicken line.

Despite the growing use of composite chicken lines in Indonesia, detailed muscle-level insights into how environmental rearing systems affect tissue morphology and physiology remain unexplored. While the IPB-D3 chicken has shown promising growth performance and adaptability, there is limited understanding of the underlying histological variations that occur under different management conditions. Detailed information on myofibre architecture, collagen organisation, and fibre-type composition, which directly influence meat quality traits, remains particularly limited. Furthermore, the interaction between rearing environment and muscle microstructure has not been systematically studied in composite local chickens, leaving a critical gap in the correlation between macroscopic growth outcomes and microscopic muscle quality determinants. Understanding these tissue-level adaptations is essential for optimizing both animal performance and consumer-oriented meat attributes in sustainable poultry production systems.

This study aimed to characterize and compare the growth performance and muscle histological features of IPB-D3 chickens reared under intensive and free-range systems. The evaluation focused on identifying structural and compositional differences in pectoralis major (Pec) and quadratus femoris (Fem) muscles, including fasciculus dimensions, myofiber density, collagen proportion, and type IIX myofiber distribution. By integrating morphometric and histochemical analyses, the study sought to elucidate how rearing systems influence muscle development patterns and potential meat quality traits in this locally developed line. The findings are intended to provide baseline histological data for genetic improvement programs and guide evidence-based recommend-dations for sustainable rearing strategies, supporting future innovations in Indonesia’s local poultry industry.

## MATERIALS AND METHODS

### Ethical approval

All experimental procedures involving animals were conducted in strict accordance with national and international guidelines for the care and use of animals in research. The study complied with the principles of the Animal Research: Reporting of *In Vivo* Experiments 2.0 guidelines, the World Organization for Animal Health Terrestrial Animal Health Code, and applicable Indonesian animal welfare regulations.

The experimental protocol was reviewed and approved by the Animal Ethics Committee of the School of Veterinary Medicine and Biomedical Sciences, IPB University, Bogor, Indonesia (Approval No. 209/KEH/SKE/ IV/2024). Ethical review covered all aspects of animal use, including housing conditions, rearing systems, feeding management, handling procedures, sampling strategy, and slaughter methods.

Throughout the study period, birds were monitored daily for health status, behavior, and signs of distress. Stocking density, ventilation, access to feed and water, and environmental enrichment were maintained to minimize stress and ensure animal welfare under both intensive and free-range systems. No invasive procedures were performed prior to slaughter, and no mortality related to experimental handling was observed.

Slaughtering was carried out at 24 weeks of age following a manual halal procedure, after a 24-h fasting period, in accordance with accepted humane slaughter practices to minimize pain and distress. Tissue sampling for histological analysis was performed only postmortem. All efforts were made to reduce the number of animals used while ensuring sufficient statistical power and scientific validity.

### Study period and location

The experiment was conducted from January to November 2024. The IPB-D3 chickens were housed at the Field Laboratory of Animal Breeding and Genetics, Faculty of Animal Science, IPB University, Dramaga, Bogor, located at approximately 250 m above sea level. Histological analyses, including hematoxylin-eosin (HE), picrosirius red (PSR), and immunohistochemical (IHC) staining of Pec and Fem muscles, were performed in the Veterinary and Zoology Laboratory, Anatomy and Histology Section, National Research and Innovation Agency (BRIN), Indonesia.

### Experimental animals and design

This preliminary study was conducted as part of the research series by Kuswandi *et al*. [31]. A total of 90 IPB-D3 chickens (12 weeks old) were used. All chickens underwent a 7-day acclimatization period before the experiment, during which their health status was monitored daily. Birds were randomly allocated into two rearing systems, intensive and free-range, each consisting of 45 chickens (21 males and 24 females), maintaining an approximately 1:1 sex ratio. The rearing period lasted 12 weeks. The IPB-D3 chicken is a fast-growing composite line derived from a cross between Pelung × Sentul (male) and Kampung × Broiler Cobb (female), each contributing 25% of the genetic composition.

### Rearing systems

#### Intensive rearing system

Forty-five chickens were raised in an enclosure measuring 6 × 1.5 m, designed to provide optimal environ-mental conditions. The housing was an open-sided conventional house without automated temperature or humidity control equipment (e.g., fans or heaters). Environmental parameters fluctuated naturally, and adequate ventilation was maintained. The flooring was constructed for easy cleaning to reduce waste accumulation and disease risk. Stocking density was adjusted to minimize stress and maintain animal welfare.

#### Free-range rearing system

Another group of 45 chickens was maintained in a 20 × 10 m free-range area containing natural vegetation such as banana trees, Indigofera, and native grasses. The area was fenced with protective netting to prevent predation and escape. The system allowed birds to express natural behaviors such as foraging, scratching, and dust bathing. Roosting supports were also provided. Chickens were released from 06:00 a.m. to 05:00 p.m., with access to shade and vegetation, and were housed indoors at night (4 × 3 m shelter) for predator protection. The rearing followed Australian free-range standards, maintaining a maximum density of one chicken per square meter [32].

#### Feeding and management

Both groups received a commercial crumble diet (B-12 L, PT. New Hope Indonesia, Indonesia) twice daily containing 13% moisture, 14%–16% crude protein, 3,000 kcal/kg metabolizable energy, 3% crude fat, 8% crude fiber, 8% ash, and 0.9%–1.2% calcium. Feed energy was derived primarily from corn, rice bran, and oil; protein sources included soybean meal, amino acids, meat, and bone meal; and minerals were provided through limestone, sodium bicarbonate, and trace minerals. Drinking water was available *ad libitum* throughout the study.

### Slaughtering and sample collection

At 24 weeks of age, chickens were slaughtered following a manual halal procedure after a 24-h fasting period. From each rearing system, five chickens (n = 10 total; 2 males and 3 females per group) were randomly selected for histological analysis. Following slaughter, carcasses were scalded in 70°C water for 10–20 s to remove feathers [33]. Internal organs were excised, and carcass weights (without giblets) were recorded. Samples of Pec and Fem muscles were collected, and 2 × 2 cm sections were fixed in 10% neutral-buffered formalin (Merck, Darmstadt, Germany) for 48 h, then transferred to 70% ethanol before standard paraffin (Merck) embedding. Tissues were sectioned serially at 5 µm thickness (three sections per muscle).

### Histological preparation and staining

#### HE staining

Sections were deparaffinized in xylene (Merck), rehydrated through graded ethanol (100%–70%, Merck), rinsed with distilled water (OneMed, Surabaya, Indonesia), and stained with HE (Merck) to examine general muscle morphology [10, 11, 34, 35]. Slides were observed using an Olympus CX43 microscope at 10×, 20×, and 40× magnifications, and images were captured under standardized brightness and white balance.

#### PSR staining

Sections were processed as above and stained with 0.1% PSR solution (Sigma Aldrich, St. Louis, USA) to evaluate intramuscular collagen distribution [36–39]. Observations were made at 10×, 20×, and 40× magnifications. Collagen content was quantified as the percentage of red–stained area per tissue field. Under polarized light, collagen fibers were categorized as thick (red-yellow), thin (green), or unpolarized/black, indicating different fiber thicknesses and types.

#### IHC staining

Sections were deparaffinized, rehydrated, and treated with 3% hydrogen peroxide (Merck) in Phosphate-buffered saline (pH 7.4, Sigma Aldrich) for 10 min, followed by 0.1%–0.2% Triton X-100 (Sigma Aldrich) permeabilization. IHC was performed using the Mouse and Rabbit Specific HRP/DAB Detection Kit (ab64264, Abcam, Cambridge, UK). After blocking, samples were incubated overnight (4°C) with anti-fast skeletal myosin heavy chains (MyHC)-2×/type IIX (ab51263, Abcam) and anti-slow skeletal MyHC-7/type I (ab11083, Abcam) monoclonal antibodies (1:60 dilution) following Huo *et al*. [23] and Weng *et al*. [40]. Visualization was achieved using DAB chromogen and substrate (ab64264, Abcam) for 2 min, followed by dehydration, clearing in xylene, and mounting with Entellan (Merck).

The staining intensity was quantified by converting images to grayscale and measuring gray values: high (144–255), moderate (50–144), and low (2–30). Fiber intensity was categorized as follows: High = type IIX, moderate = mixed type IIX, and low = other myofiber types.

#### Image analysis

Quantitative analyses were conducted using ImageJ software (v1.53k, NIH, USA). Calibration was performed with a micrometer (Erma, Tokyo, Japan).

- For HE-stained sections, the following were measured: Fasciculus area (average of 10), myofiber cross-sectional area (average of 60), and myofiber number per mm^2^ (five fields per sample).
- For PSR staining, the relative percentage of intramuscular collagen was analyzed in five random fields per muscle (10× magnification).
- For IHC staining, the percentage of type IIX myofibers was determined across five fields per sample.

#### Statistical analysis

Data were tested for normality (Shapiro-Wilk) and homogeneity of variances (Levene’s test) before analysis. Parameters that satisfied parametric assumptions were compared using the independent t-test at a 95% confidence level (α = 0.05). Statistical analyses were performed using the Statistical Package for the Social Sciences v.29.0 (IBM, New York, USA).

## RESULTS

### Growth performance

The growth performance of IPB-D3 chickens did not differ significantly (p > 0.05) between the two rearing systems (Table 1). Both intensive and free-range chickens exhibited comparable body and carcass weights, indicating similar growth efficiency under different management conditions.

**Table 1:** Growth performance parameters of IPB-D3 chickens reared under intensive and free-range production systems.

Parameter (g)	Reared systems	

	Intensive	Free-range
Body weight	1222.40 ± 206.04	1340.20 ± 322.48
Carcass weight	801.60 ± 163.10	854.00 ± 218.20
Breast weight	213.43 ± 57.83	199.71 ± 41.32
Thigh weight	65.36 ± 16.85	68.05 ± 19.57

Values are expressed as mean ± standard error of the mean.

### Muscle microstructure and histomorphometry

Microscopic examination revealed that the myofiber cross-sectional area of the Fem muscle was significantly larger in chickens reared under the free-range system compared with those reared intensively (Hedges’ g = 1.088; 95% confidence interval [CI] [0.175, 2.296]; p < 0.05) (Table 2). Likewise, the proportion of high-intensity type IIX myofibers was greater in the Fem muscle of free-range chickens (Hedges’ g = 11.657; 95% CI [−0.170, 2.610]; p < 0.05) (Table 3). In contrast, the relative percentage of intramuscular collagen was higher in the Fem muscle of intensively reared chickens (Hedges’ g = 1.329; 95% CI [−2.668, −0.058]; p < 0.05) (Table 2).

**Table 2:** Histomorphometric characteristics of IPB-D3 chicken muscles under intensive and free-range rearing systems.

Parameters	Rearing system	

	Intensive	Free-range
Fasciculus area (10^5^× µm^2^)		
Fem	1.33 ± 0.08	1.39 ± 0.12
Pec	1.95 ± 0.15*	1.83 ± 0.80*
Myofiber cross-sectional area (10^3^× µm^2^)		
Fem	1.91 ± 0.37*	2.39 ± 0.42*^,α^
Pec	1.21 ± 0.29	1.30 ± 0.39
Number of myofibers per mm^2^		
Fem	232.65 ± 42.16	215.03 ± 98.90
Pec	467.18 ± 77.90*	373.28 ± 82.58*
Relative percentage of collagen (%)		
Fem	8.92 ± 1.34*^,α^	7.07 ± 1.04
Pec	5.73 ± 1.72	7.43 ± 1.45

Values are presented as mean ± standard deviation.

* indicates a significant difference between Pec and Fem within the same rearing system (same column) (p < 0.05), α indicates a significant difference between intensive and free-range systems within the same muscle type (same row) (p < 0.05). Units are expressed as indicated for each parameter. Fem = Quadratus femoris muscle, Pec = Pectoralis major muscle.

**Table 3:** Distribution (%) of type IIX myofibers in IPB-D3 chicken muscles under intensive and free-range rearing systems based on immunostaining intensity.

Immunostaining intensity	Intensive		Free-range	
	
	Fem	Pec	Fem	Pec
High (%)	19.03 ± 9.23*	2.95 ± 1.77	33.69 ± 10.95*^,α^	6.13 ± 5.25
Moderate (%)	67.81 ± 14.46	92.95 ± 3.78*	54.29 ±14.51	88.03 ± 4.73*
Low (%)	13.17 ± 8.41	4.10 ± 3.48	12.03 ±11.22	5.78 ± 5.11

Values are expressed as mean ± standard deviation.

* Indicates a significant difference between Fem and Pec within the same rearing system (p < 0.05), α indicates a significant difference between intensive and free-range systems within the same muscle type (same row) (p < 0.05). Fem = Quadratus femoris muscle, Pec = Pectoralis major *muscle*. Immunostaining intensity was classified as high, moderate, or low based on staining strength.

**Figure 1:**
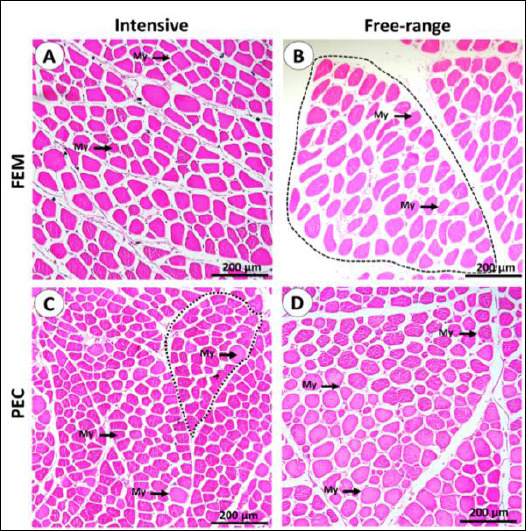
General histological architecture of IPB D-3 chicken muscle. (A–D) Representative hematoxylin and eosin–stained sections of muscles from different rearing systems, illustrating muscle fascicles (dashed outlines) and individual myofibers (My). Fem = Quadratus femoris muscle, Pec = Pectoralis major muscle.

**Figure 2:**
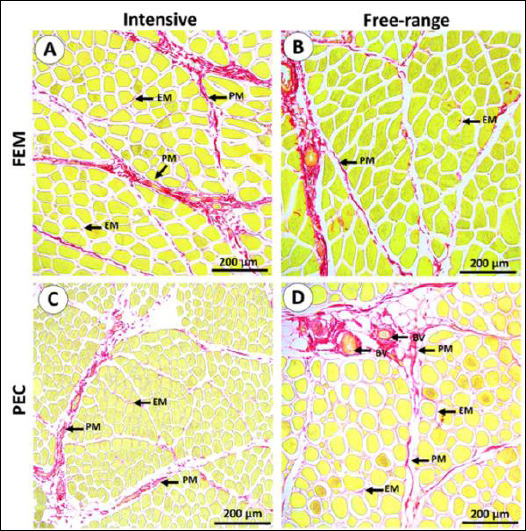
(A–D) Representative picrosirius red–stained sections highlighting intramuscular connective tissue (red) in different muscles from distinct rearing systems. The perimysium (PM) and endomysium (EM) are clearly delineated within the muscle tissue. Fem = Quadratus femoris muscle, Pec = Pectoralis major muscle.

**Figure 3:**
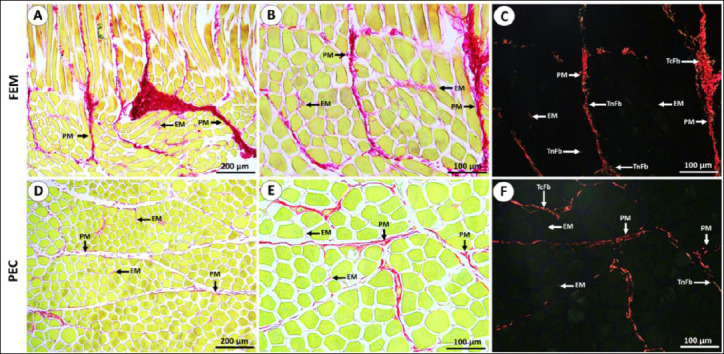
**(A–C)** Representative picrosirius red–stained sections showing collagen distribution in the femoral (Fem) muscle of IPB-D3 chickens, and **(D–F)** corresponding sections of the pectoralis (Pec) muscle. Collagen is observed as discontinuous fibers within the perimysium (PM), while the endomysium (EM) contains finer fibers than the perimysium. **(C, F)** Polarized light micrographs demonstrate that the perimysium predominantly comprises thick collagen fibers (TcFb) with some thin fibers (TnFb), whereas the EM appears weakly or non-polarized, indicating the presence of very thin fibers or different collagen types. Fem = Quadratus femoris muscle, Pec = Pectoralis major muscle.

Overall, no distinct morphological abnormalities or fiber arrangement differences were observed between the two rearing systems. The muscle microstructure of IPB-D3 chickens consisted of fasciculi bound by collagenous perimysium, with individual myofibers enclosed by endomysium (EM). Most myofibers displayed a polygonal shape, although some appeared round (Figures 1 and 2). Under polarized light, thick collagen fibers were predominantly localized in the perimysium, while thin fibers were observed mainly in the EM (Figure 3).

### Comparative muscle histomorphometry

Although overall morphology appeared similar, two histomorphometric parameters differed significantly (p < 0.05) between rearing systems. The Pec muscle contained more myofibers per mm^2^ than the Fem muscle, corresponding to its smaller fiber size. Conversely, the Fem muscle in free-range chickens exhibited larger fasciculus and myofibril areas, reflecting muscle hypertrophy associated with higher physical activity. In both rearing systems, the fasciculus area was significantly greater (p < 0.05) in Pec than in Fem muscles. Within the intensive system, the relative intramuscular collagen percentage was significantly higher in Fem than in Pec muscles (Table 2).

### IHC localization of type IIX myofibers

**Figure 4:**
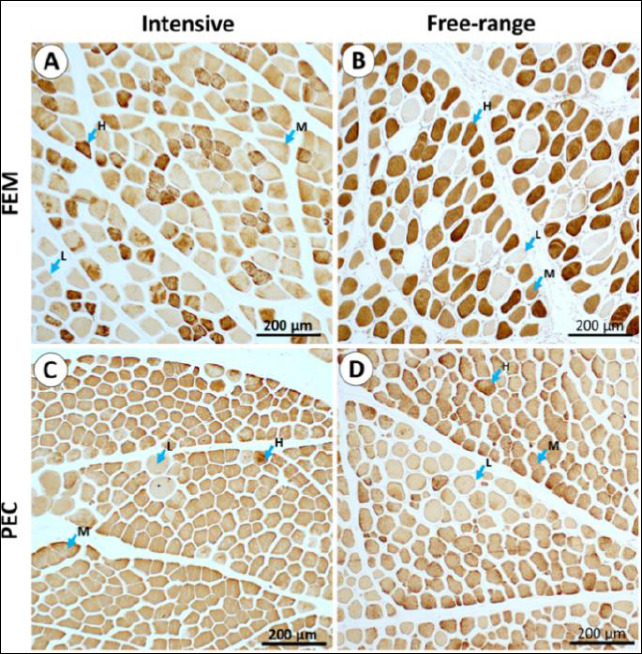
**(A and B)** Immunohistochemical localization of type IIX myofibers in the femoral (Fem) muscle and **(C and D)** in the pectoralis (Pec) muscle across different rearing systems. Myofibers exhibit variable staining intensity, categorized as high (H), moderate (M), or low (L), reflecting differences in antibody binding to type IIX myofibers. Fem = Quadratus femoris muscle, Pec = Pectoralis major muscle.

IHC analysis revealed clear variations in staining intensity among muscle fibers, representing differential antibody binding to type IIX MyHC. The distribution patterns of type IIX fibers varied between Fem and Pec muscles (Figure 4) but remained generally consistent across rearing systems (Table 3).

**Figure 5:**
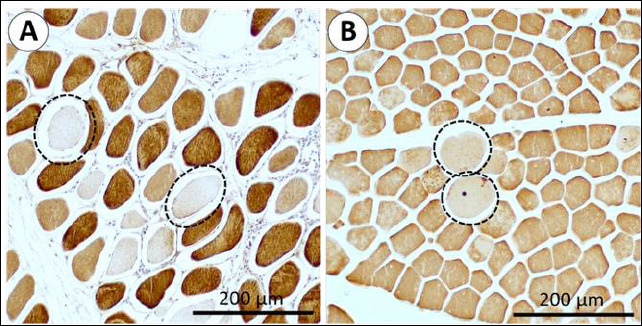
**(A)** Femoral and **(B)** pectoralis muscle sections showing low-intensity immunohistochemical staining localized to round-shaped myofibers (circled with dotted lines).

High-intensity myofibers, corresponding to type IIX fibers, were more abundant in the Fem muscle and particularly prevalent in free-range chickens. Moderate-intensity fibers were more frequently observed in the Pec muscle, whereas low-intensity fibers showed high variability with no significant differences between muscle types or rearing systems (Table 3). Morphologically, low-intensity fibers exhibited a round shape, while high-intensity fibers appeared polygonal (Figure 5).

Notably, IHC for slow-type I myofibers yielded negative results in all samples, indicating a predominance of fast-twitch fiber composition in the muscles of IPB-D3 chickens.

## DISCUSSION

### Growth performance of IPB-D3 chickens under different rearing system

No significant differences were observed in the body growth performance of IPB-D3 chickens between the intensive and free-range systems. This outcome is consistent with the genetic potential of the IPB-D3 line, which is a fast-growing composite breed derived from IPB-D1 chickens registered under the Ministry of Agriculture of the Republic of Indonesia (Decree No. 693/KPTS/PK.230/M/9/2019). The parent line, IPB-D1, is known for its adaptability to both intensive and free-range systems, showing efficient feed conversion ratios of approximately 2.88 (intensive) and 3.44 (free-range) [41]. The comparable performance of IPB-D3 chickens in both rearing systems indicates a stable growth efficiency and adaptability of this improved local line across diverse management conditions.

### Effect of rearing systems on muscle microstructure

The significant increase in the myofiber cross-sectional area of the Fem muscle in free-range chickens suggests enhanced muscle activity and hypertrophy. This effect can be attributed to greater locomotor activity and environmental stimulation in the free-range system, which promotes muscle fiber enlargement. Similar findings were reported by Fu *et al*. [42], where the thigh muscle diameter was significantly higher in free-range chickens than in caged counterparts at 26 weeks of age. Free-range conditions have also been associated with increased thigh muscle development in Lueyang black-bone chickens [43]. However, since the initial myofiber size at the start of this study was not measured, longitudinal evaluation across growth stages is recommended for future work.

### Comparative myofiber dimensions among chicken breeds

The current study provides the first characterization of myofibers in IPB-D3 chickens. The Pec fasciculus area of IPB-D3 (1.83–1.95 × 10^5^ µm^2^) was slightly smaller than that of Pelung chickens (2.1 × 10^5^ µm^2^), one of its parental lines. Similarly, the cross-sectional area of breast myofibers in IPB-D3 chickens (≈1210–1300 µm^2^) was smaller than that of mature Pelung chickens (1937.74 µm^2^ at 40–56 weeks, body weight ≈3 kg) [11]. Kampung chickens at 63 days (924 g) had smaller myofibers (960.56 µm^2^) [15], while broilers aged 21 days and weighing 48 g showed even smaller fiber areas, ranging from 310.02 to 769.59 µm^2^ [13, 14, 22]. In Sentul chickens (12 weeks old), thigh and breast myofiber diameters were 27.74 µm^2^ and approximately 39 µm^2^, respectively. These comparisons highlight the broad range of myofiber dimensions across chicken breeds, influenced by genotype, age, nutrition, and muscle type [44]. Since meat quality is closely related to histological and biochemical muscle properties [45, 46], understanding fiber size variation is critical. Larger myofibers generally correlate with reduced tenderness due to denser muscle texture [47].

### Myofiber number and collagen distribution

The Pec muscle contained more myofibers per mm^2^ than the Fem muscle, reflecting smaller fiber dimensions. According to Scheuermann *et al*. [48] and Huo *et al*. [49], increased fiber number contributes to higher breast meat yield, although fiber hypertrophy remains the dominant determinant of total muscle mass. Other biological factors such as bone growth and fat deposition may also influence body weight, as noted by Lawrence and Fowler [50] and Li *et al*. [51].

Histological observations revealed discontinuous intramuscular collagen fibers within the perimysium, predominantly composed of thick fibers, while the EM consisted mainly of thin fibers. In the intensive rearing system, the Fem muscle contained a higher proportion of intramuscular collagen than its free-range counterpart. Variability in collagen deposition among samples suggests its contribution to meat texture and tenderness [52]. Thickened perimysial connective tissue is known to increase shear force values in various meats, including chicken, whereas higher intramuscular fat content generally decreases shear force [53–55]. These findings indicate that collagen composition, though important, is only one of several factors determining overall meat tenderness.

### Type IIX myofiber characteristics and meat quality implications

High-intensity type IIX myofibers were more abundant in the Fem muscle of free-range chickens, suggesting rearing-related differences in fiber-type distribution. Muscles with a predominance of type IIX fibers tend to exhibit poorer meat quality, characterized by paler color, reduced water-holding capacity, and lower tenderness, primarily due to enhanced glycolytic metabolism and a rapid postmortem pH decline [56–58]. The larger myofiber cross-sectional area observed in the Fem muscle further supports the hypertrophy of fast-twitch fibers under active movement. Du *et al*. [59] similarly reported that fast-growing chickens possess larger myofiber diameters and a greater proportion of type II fibers than slow-growing breeds. Type IIX fibers, identified as high-intensity white fibers with low myoglobin content, are typically associated with coarser meat texture [60]. Intermediate-intensity fibers may represent mixed fiber types, as seen in Ross 308 broilers and Xueshan chickens [23, 40, 61]. Such heterogeneity indicates the adaptive plasticity of skeletal muscles, where environmental and physiological stimuli can induce fiber-type shifts [62–64]. The higher occurrence of type IIX fibers in the Fem muscle of free-range IPB-D3 chickens may therefore influence the textural properties of thigh meat and warrants further biochemical and sensory evaluation.

### Morphological variation and myofiber maturity

The present study also noted that low-intensity stained myofibers were predominantly round, whereas high- and moderate-intensity fibers exhibited polygonal shapes in both Pec and Fem muscles. Rounded fibers with variable diameters are often indicative of regenerating or immature fibers, whereas polygonal fibers represent mature, functionally stable myofibers [65, 66]. Soendenbroe *et al*. [61] associated round myofibers with type I fibers, consistent with the current observation that round fibers in IPB-D3 chickens showed low IHC intensity for type IIX. However, all samples were negative for slow-type I myofiber staining, despite positive control reactivity in Ross 308 and Xueshan chickens [23, 40]. This suggests that the IPB-D3 line predominantly consists of fast-twitch fibers, though further molecular confirmation is required.

## CONCLUSION

This study provides the first comprehensive evaluation of the growth performance and muscle histological characteristics of the IPB-D3 chicken under intensive and free-range rearing systems. The findings revealed that overall growth performance did not differ significantly (p > 0.05) between systems, confirming the adaptability of IPB-D3 chickens to both management conditions. However, free-range rearing significantly increased the myofiber cross-sectional area and the proportion of high-intensity type IIX fibers in the Fem muscle (*p* < 0.05), suggesting enhanced muscle hypertrophy due to increased physical activity. Conversely, intensively reared chickens exhibited higher intramuscular collagen deposition, particularly in thigh muscles, which could affect meat tenderness. The Pec muscle contained a greater myofiber density per mm², reflecting its smaller fiber size and higher yield potential.

From a practical standpoint, these results indicate that IPB-D3 chickens can thrive efficiently in both intensive and free-range systems, offering flexibility for smallholders and commercial producers. Free-range rearing enhances muscle development and supports animal-welfare-oriented production, while intensive rearing facilitates better carcass uniformity and improved management efficiency. These findings contribute to the development of sustainable poultry systems that balance productivity, meat quality, and welfare – aligning with Sustainable Development Goal (SDG 2; Zero Hunger) and SDG 12 (Responsible Consumption and Production).

The strength of this study lies in its integrated histomorphometric and IHC approach, which provides baseline data for muscle fiber-type distribution and collagen structure in IPB-D3 chickens, a parameter previously unreported in Indonesian composite breeds. However, certain limitations should be acknowledged: unequal sex ratios, limited sample size, absence of longitudinal sampling, and lack of analyses of meat texture and gene expression.

Future studies should incorporate molecular assays for MyHC isoforms, texture and sensory evaluations, and growth-stage comparisons to better elucidate muscle plasticity and quality dynamics. Long-term assessments of nutritional interventions and rearing environments are also warranted to optimize the IPB-D3 line for broader production contexts.

In conclusion, the present findings underscore the dual potential of the IPB-D3 chicken as a robust, adaptable, and fast-growing local breed suitable for both intensive and free-range systems, providing a valuable foundation for advancing sustainable, welfare-friendly poultry production in Indonesia.

## DATA AVAILABILITY

The data that support the findings of this study are available from the corresponding author upon reasonable request.

## AUTHORS’ CONTRIBUTIONS

AYP, RFY, IK, AF, DL, NLPRP, CB, and CS: Planned the study, designed the experiment, recorded, proofread, and revised the manuscript. AYP, NLPRP, and RFY: Analyzed samples. AYP and RFY: Drafted the manuscript. All authors have read and approved the final version of the manuscript.

## References

[1] Adelta KB, Arief II, Sumantri C, Wulandari Z (2023). Meat quality characteristics of IPB-D1 chicken and the final stock from different locations. J Ilmu Ternak Vet.

[2] Dal Bosco A, Mattioli S, Cartoni Mancinelli A, Cotozzolo E, Castellini C (2021). Extensive rearing systems in poultry production:The right chicken for the right farming system. A review of twenty years of scientific research in Perugia University, Italy. *Animals (Basel).*.

[3] Bakrie B, Rohaeni ES, Yusriani Y, Tirajoh S (2021). The development of a newly formed superior local chicken in Indonesia-a review. J Hunan Univ Nat Sci.

[4] Besbes B (2009). Genotype evaluation and breeding of poultry for performance under sub-optimal village conditions. World's Poult Sci J.

[5] Bekele G, Aleme M (2023). A review:Production and reproduction performance of cross bred chicken in Ethiopia. Poult Fish Wild Sci.

[6] Sumantri C, Khaerunnisa I, Gunawan A (2020). The genetic quality improvement of native and local chickens to increase production and meat quality in order to build the Indonesian chicken industry. IOP Conf Ser Earth Environ Sci.

[7] Rahman WN, Darwati S, Sumantri C (2024). Comparison of carcass and non-carcass weight percentage between IPB-D2 chickens and IPB-D3 chickens at different slaughter age. IOP Conf Ser Earth Environ Sci.

[8] El-Deek A, El-Sabrout K (2019). Behaviour and meat quality of chicken under different housing systems. World's Poult Sci J.

[9] Sidiqi AAA, Airin CM, Sarmin S, Astuti P (2023). A combination of *Anadara nodifera* shell and milkfish thorns powder effectively promote springiness index, serum testosterone, and breast muscle testosterone in Bangkok rooster. Hayati J Biosci.

[10] Yuneldi RF, Airin CM, Saragih HT, Sarmin S, Astuti P, Alimon AR (2023). Growth, pectoralis muscle performance, and testis of Pelung cockerels (*Gallus gallus gallus* [Linnaeus, 1758]) supplemented with blood clam shell powder (*Anadara granosa* [Linnaeus, 1758]).. Vet World.

[11] Yuneldi RF, Airin CM, Saragih HTS, Prawira AY, Astuti P (2024). Testosterone hormone levels and breast muscle performance of Pelung chickens after zinc sulfate and synthetic testosterone supplementation. Vet World.

[12] Murtini S, Gunawan A, Khaerunnisa I, Lestari D, Fastawa R, Anggraeni A, Kim YS, Sumantri C (2025). Effects of maternal antibodies against myostatin on post-hatch chicken growth and muscle mass in Sentul Indonesian indigenous chicken. Vet World.

[13] Blatama D, Salsabila N, Saragih HT (2023). *Hornstedtia scottiana* fruit as a feed additive to improve the histological structures and growth performance of broiler. Vet World.

[14] Saragih HT, Fauziah IN, Saputri DA, Chasani AR (2024). Dietary macroalgae *Chaetomorpha linum* supplementation improves morphology of small intestine and pectoral muscle, growth performance, and meat quality of broilers. Vet World.

[15] Saragih HTSSG, Salsabila N, Deliaputri R, Firdaus ABI, Kurnianto H (2024). Growth morphology of the gastrointestinal tract, *pectoralis thoracicus* muscle, lymphoid organ and visceral index of kampong chicken. J Saudi Soc Agric Sci.

[16] Zhang D, Xu F, Liu Y (2024). Research progress on regulating factors of muscle fiber heterogeneity in poultry:A review. Poult Sci.

[17] Ismail I, Joo ST (2017). Poultry meat quality in relation to muscle growth and muscle fiber characteristics. Korean J Food Sci Anim Resour.

[18] Petracci M, Sirri F, Mazzoni M, Meluzzi A (2013). Comparison of breast muscle traits and meat quality characteristics in 2 commercial chicken hybrids. Poult Sci.

[19] Choi YM, Kim BC (2009). Muscle fiber characteristics, myofibrillar protein isoforms, and meat quality. Livest Sci.

[20] Wu X, Zhou X, Ding X, Chu M, Liang C, Pei J, Xiong L, Bao P, Guo X, Yan P (2020). Reference gene selection and myosin heavy chain (MyHC) isoform expression in muscle tissues of domestic yak (*Bos grunniens*). PLoS One.

[21] Xi Y, Liu H, Zhao Y, Li J, Li W, Liu G, Lin J, Liu W, Zhang J, Lei M, Ni D (2018). Comparative analyses of longissimus muscle miRNAomes reveal microRNAs associated with differential regulation of muscle fiber development between Tongcheng and Yorkshire pigs. PLoS One.

[22] Saragih HT, Susanto A, Aditya NC, Damayanti SAC, Firdaus ABI, Salsabila N, Nuriliani A (2024). Growth performance of broiler chicken supplemented with water-extracted red dragon fruit (*Hylocereus polyrhizus*). J Ilmu Ternak Vet.

[23] Huo W, Weng K, Li Y, Zhang Y, Zhang Y, Xu Q, Chen G (2022). Comparison of muscle fiber characteristics and glycolytic potential between slow- and fast-growing broilers. Poult Sci.

[24] Hawari MF, Sumantri C, Darwati S (2024). Egg production and quality of IPB D3 chicken and its repeatability estimation. J Ilmu Prod Teknol Has Peternak.

[25] Galib I, Sumantri C, Darwati S, Murtini S (2024). Weight performance of 4th generation IPB-D3 local chickens aged 1–3 months and its heritability value. J Ilmu Prod Teknol Has Peternak.

[26] Rizqi MA, Sumantri C, Darwati S (2025). Growth performance of IPB D2 with IPB D3 chicken line crossing and its reciprocal. IOP Conf Ser Earth Environ Sci.

[27] Ratnawati D, Darwati S, Murtini S, Sumantri C (2025). Productivity of IPB-D2 and IPB-D3 chickens with repeatability of Newcastle disease antibody titer. J Ilmu Pertan Indonesia.

[28] Romantis S, Sumiati S, Sumantri C (2024). Effects of supplementation with different nutrient contents of vitamin E diets on the performance and health status of IPB-D3 candidate chicken strains. J Ilmu-Ilmu Peternak.

[29] Rizqi MA, Sumantri C, Darwati S (2024). Morphometrics of IPB D1, IPB D2 and IPB D3 chickens aged 4 to 12 weeks at Sinar Harapan Farm, Sukabumi. J Ilmu Prod Teknol Has Peternak.

[30] Vianisa P, Murtini S, Furqon A, Sumantri C (2025). Immunocompetence index performance of the IPB-D3 candidate line under different rearing systems. J Ilmu-Ilmu Peternak.

[31] Kuswandi W, Budiman C, Khaerunnisa I, Sumantri C (2025). Rearing system and immune status influence the small intestinal microbiota of IPB-D3 chickens:A full-length 16S rRNA metagenomic approach. Vet World.

[32] McCormack M (2017). Australian Consumer Law (Free-range Egg Labelling) Information Standard 2017. Minister for Small Business, Australia.

[33] Mrajji O, Wazna ME, Boussoualem Y, Bouari AE, Cherkaoui O (2021). Feather waste as a thermal insulation solution:Treatment, elaboration and characterization. J Ind Text.

[34] Saragih HT, Muhamad AAK, Alfianto A, Viniwidihastuti F, Untari LF, Lesmana I, Widyatmoko H, Rohmah Z (2019). Effects of *Spirogyra jaoensis* as a dietary supplement on growth, pectoralis muscle performance, and small intestine morphology of broiler chickens. Vet World.

[35] Yuneldi RF, Saraswati TR, Yuniwarti EYW (2021). The histomorphometry of liver and kidney of hyperglycemic albino rats after treatment with *Tithonia diversifolia* leaf extract. Biosaintifika J Biol Biol Educ.

[36] Prawira AY, Novelina S, Farida WR, Darusman HS, Warita K, Hosaka YZ, Agungpriyono S (2022). Determination of thick and thin fibres distribution in Sunda porcupine dorsal skin (*Hystrix javanica*) using picrosirius red staining. Anat Histol Embryol.

[37] Prawira AY, Supratikno, Anwar S, Khaerunnisa I, Furqon A, Novelina S, Prihatin KW (2024). Preliminary examination of the histochemistry of the semitendinosus muscle microstructure in Bali cattle (*Bos javanicus*) and the correlations with muscle score. Anat Histol Embryol.

[38] Prawira AY, Hosaka YZ, Novelina S, Farida WR, Darusman HS, Agungpriyono S (2020). Morphological evaluation of polysaccharide content and collagen composition during cutaneous wound healing in the Sunda porcupine (*Hystrix javanica*). J Vet Med Sci.

[39] Prawira AY, Phadmacanty NLPR, Semiadi G, Kurniati H, Trilaksono W, Agungpriyono S (2024). Water monitor lizard (*Varanus salvator*) skin microstructure:Histochemical and morphometrical studies of fiber-type characteristics. Hayati J Biosci.

[40] Weng K, Huo W, Li Y, Zhang Y, Zhang Y, Chen G, Xu Q (2022). Fiber characteristics and meat quality of different muscular tissues from slow- and fast-growing broilers. Poult Sci.

[41] Al-Habib MF, Murtini S, Cyrilla L, Arief II, Mutia R, Sumantri C (2020). The growth performance of IPB-D1 chickens in different feed treatments and production systems. J Agripet.

[42] Fu D, Zhang D, Xu G, Li K, Wang Q, Zhang Z, Li J, Chen Y, Jia Y, Qu L (2015). Effects of different rearing systems on meat production traits and meat fiber microstructure of Beijing-you chicken. Anim Sci J.

[43] Cheng J, Wang L, Wang S, Chen R, Zhang T, Ma H, Lu H, Yuan G (2023). Transcriptomic analysis of thigh muscle of Lueyang black-bone chicken in free-range and caged feeding. Anim Biotechnol.

[44] Honda M, Tsuchimochi H, Hitachi K, Ohno S (2019). Transcriptional cofactor Vgll2 is required for functional adaptations of skeletal muscle induced by chronic overload. J Cell Physiol.

[45] Rehfeldt C, Fiedler I, Dietl G, Ender K (2000). Myogenesis and postnatal skeletal muscle cell growth as influenced by selection. Livest Prod Sci.

[46] Wegner J, Albrecht E, Fiedler I, Teuscher F, Papstein HJ, Ender K (2000). Growth- and breed-related changes of muscle fibre characteristics in cattle. J Anim Sci.

[47] Joo SH, Lee KW, Hwang YH, Joo ST (2017). Histochemical characteristics in relation to meat quality traits of eight major muscles from Hanwoo steers. Korean J Food Sci Anim Resour.

[48] Scheuermann GN, Bilgili SF, Tuzun S, Mulvaney DR (2004). Comparison of chicken genotypes:Myofiber number in pectoralis muscle and myostatin ontogeny. Poult Sci.

[49] Huo W, Weng K, Gu T, Zhang Y, Zhang Y, Chen G, Xu Q (2021). Effect of muscle fiber characteristics on meat quality in fast- and slow-growing ducks. Poult Sci.

[50] Lawrence TLJ, Fowler VR (2002). Growth of Farm Animals.

[51] Li Z, Mushtaq M, Khan M, Fu J, Rahman A, Long Y, Liu Y, Zi X, Sun D, Ge C, Wang K (2024). Evaluation of the growth performance and meat quality of different F1 crosses of Tengchong snow and Xichou Blackbone chicken breeds. Animals (Basel).

[52] Purslow PP (2020). The structure and role of intramuscular connective tissue in muscle function. Front Physiol.

[53] Purslow PP (2005). Intramuscular connective tissue and its role in meat quality. Meat Sci.

[54] Roy BC, Bruce HL (2023). Contribution of intramuscular connective tissue and its structural components on meat tenderness-revisited:A review. Crit Rev Food Sci Nutr.

[55] Roy BC, Das C, Aalhus JL, Bruce HL (2021). Relationship between meat quality and intramuscular collagen characteristics of muscles from calf-fed, yearling-fed and mature crossbred beef cattle. Meat Sci.

[56] Mo M, Zhang Z, Wang X, Shen W, Zhang L, Lin S (2023). Molecular mechanisms underlying the impact of muscle fiber types on meat quality in livestock and poultry. Front Vet Sci.

[57] Song S, Ahn CH, Song M, Kim GD (2020). Pork loin chop quality and muscle fiber characteristics as affected by the direction of cut. Foods.

[58] Matarneh SK, Silva SL, Gerrard DE (2021). New insights in muscle biology that alter meat quality. Annu Rev Anim Biosci.

[59] Du YF, Ding QL, Li YM, Fang WR (2017). Identification of differentially expressed genes and pathways for myofiber characteristics in soleus muscles between chicken breeds differing in meat quality. Anim Biotechnol.

[60] Murach KA, Dungan CM, Kosmac K, Voigt TB, Tourville TW, Miller MS, Bamman MM, Peterson CA, Toth MJ (2019). Fiber typing human skeletal muscle with fluorescent immunohistochemistry. J. Appl. Physiol.

[61] Soendenbroe C, Karlsen A, Svensson RB, Kjaer M, Andersen JL, Mackey AL (2024). Marked irregular myofiber shape is a hallmark of human skeletal muscle ageing and is reversed by heavy resistance training. J Cachexia Sarcopenia Muscle.

[62] Schiaffino S, Reggiani C (2011). Fiber types in mammalian skeletal muscles. Physiol Rev.

[63] Schiaffino S, Gorza L, Sartore S, Saggin L, Ausoni S, Vianello M, Gundersen K, Lømo T (1989). Three myosin heavy chain isoforms in type 2 skeletal muscle fibres. J Muscle Res Cell Motil.

[64] Quiat D, Voelker KA, Pei J, Grishin NV, Grange RW, Bassel-Duby R, Olson EN (2011). Concerted regulation of myofiber-specific gene expression and muscle performance by the transcriptional repressor Sox6. Proc Natl Acad Sci USA.

[65] Desgeorges T, Liot S, Lyon S, Bouvière J, Kemmel A, Trignol A, Rousseau D, Chapuis B, Gondin J, Mounier R, Chazaud B, Juban G (2019). Open-CSAM, a new tool for semi-automated analysis of myofiber cross-sectional area in regenerating adult skeletal muscle. Skelet Muscle.

[66] Collins BC, Shapiro JB, Scheib MM, Musci RV, Verma M, Kardon G (2024). Three-dimensional imaging studies in mice identify cellular dynamics of skeletal muscle regeneration. Dev Cell.

